# Degradation of Iodinated Contrast Media in Aquatic Environment by Means of UV, UV/TiO_2_ Process, and by Activated Sludge

**DOI:** 10.1007/s11270-015-2383-9

**Published:** 2015-04-17

**Authors:** Ewa Borowska, Ewa Felis, Sebastian Żabczyński

**Affiliations:** 1 Environmental Biotechnology Department, The Silesian University of Technology, Akademicka 2, 44-100 Gliwice, Poland; 2 Centre for Biotechnology, The Silesian University of Technology, Krzywoustego 8, 44-100 Gliwice, Poland

**Keywords:** Iodinated contrast media, Membrane bioreactors, Photolysis, UV/TiO_2_ process

## Abstract

Iodinated contrast media (ICM), which are used for radiological visualization of human tissue and cardiovascular system, are poorly biodegradable; hence, new methods of their removal are sought. In this study, the effectiveness of selected X-ray ICM removal by means of UV and UV/TiO_2_ pretreatment processes from synthetic hospital wastewater was demonstrated. The following compounds were investigated: iodipamide, iohexol, and diatrizoate. The experiments were as follows: (i) estimated susceptibility of the ICM to decay by UV radiation in different aquatic matrices, (ii) determined an optimal retention time of hospital wastewater in the UV reactor, (iii) determined optimum TiO_2_ concentration to improve the effectiveness of the UV pretreatment, and (iv) investigated removal of ICM by combination of the photochemical and biological treatment methods. The quantum yields of selected ICM decay in deionized water (pH = 7.0) were established as 0.006, 0.004, and 0.029 for iohexol, diatrizoate, and iodipamide, respectively. Furthermore, the experiments revealed that diatrizoate and iohexol removal in the UV/TiO_2_ process is more efficient than in UV process alone. For diatrizoate, the removal efficiency equaled to 40 and 30 %, respectively, and for iohexol, the efficiency was 38 and 27 %, respectively. No significant increase in iodipamide removal in UV and UV/TiO_2_ processes was observed (29 and 28 %, respectively). However, highest removal efficiency was demonstrated in synthetic hospital wastewater with the combined photochemical and biological treatment method. The removal of diatrizoate and iohexol increased to at least 90 %, and for iodipamide, to at least 50 %.

## Introduction

The occurrence and ecotoxicological effects of the pharmaceuticals in the aquatic environment have been defined as one of the most emerging problems in the environmental chemistry (Daughton and Ternes 1999; Halling-Sorensen et al. 1998; Heberer 2002; Kummerer 2001, 2009). More than 80 pharmaceutically active compounds, including iodinated X-ray contrast media, have been detected up to the microgram per liter levels in sewage, surface, and ground waters (Heberer 2002; Mompelat et al. 2009). Iodinated X-ray contrast media (ICM) are frequently applied in clinical diagnosis for imaging soft tissues such as blood vessels and organs (Christiansen 2005). The structure of ICM is based on a benzene ring containing three iodine substituents which increase X-ray absorption and thereby allow for visualization of the organ or tissue. Furthermore, these polar atoms ensure high water solubility. However, their chemical structure is very stable in human organisms, and thus, ICM are excreted via urine mostly in unmetabolized form (Seitz et al. 2006). Taking in account the high numbers of X-ray examinations performed each year, this results in high volumes of ICM compounds released into the environment. For example, in Germany alone, approximately 500 t annum^−1^ of ICM are applied (Schulz et al. 2008), with hospital wastewater as the main source of ICM (Knodel et al. 2011).

It has been shown that ICM could not be eliminated completely by the conventional wastewater treatment processes, which results in their discharge into the aquatic environment (Perez and Barcelo 2007; Putschew et al. 2000; Ternes and Hirsch 2000). The ICM has been detected in many types of aquatic matrices. For example, in Germany, the concentration levels of diatrizoate, iopromide, and iomeprol frequently exceeded 1 μg L^−1^ in the influent and the effluent of a municipal wastewater treatment plant (WWTP) (Ternes and Hirsch 2000).

Putschew et al. (2000) detected ICM in all water bodies, i.e., WWTP influents and effluents, surface water, as well as in bank filtrate and drinking water. Thus, in all countries with developed medical care system, ICM are expected to be present at appreciable quantities in sewage effluence and eventually in receiving waters. Their stable chemical structure results in persistence in the environment (Heberer 2002). Few ecotoxicological studies on toxicity of selected ICM showed that their release into wastewater and surface water is not expected to pose a threat to the aquatic environment (Steger-Hartmann et al. 1999, 2002; Haiss and Kummerer 2006). Nevertheless, high concentration of ICM in the aquatic environment and their highly persistent nature cannot be ignored. Ubiquitous presence of ICM and their degradation products became a reason for monitoring of these substances in the aquatic environment (Seitz et al. 2006).

In order to minimize the amount of ICM discharged into the aquatic environment, new methods of their degradation are investigated. Apart from biological methods, chemical techniques seem to be an attractive option. In particular, photochemical processes seem suitable due to their high effectiveness in pharmaceuticals removal from wastewater (Klavarioti et al. 2009). Among these methods, ICM degradation was already investigated in the advanced oxidation processes (involving reactive species) such as UV/H_2_O_2_, UV/TiO_2_, and O_3_/H_2_O_2_ (Doll and Frimmel 2004; Huber et al. 2005; Ternes et al. 2003). Doll and Frimmel (2003) investigated photolysis of ICM by stimulated solar UV radiation. Ternes et al. (2003) compared the efficiency of the ozonolysis and advanced oxidation processes (O_3_/UV-low pressure mercury lamp, O_3_/H_2_O_2_) for ICM removal from municipal wastewater. However, in their study, usage of ozone in the concentration range of 10–15 mg L^−1^ did not result in complete ICM removal, and the advanced oxidation processes mentioned above did not cause significantly higher ICM removal than ozone alone. Huber et al. (2005) also concluded that ozonation itself was insufficient for complete elimination of ICM from wastewater. Ning et al. (2009) combined ozone with ultrasound irradiation treatment and reported almost complete decay of selected X-ray contrast media, while Kwon et al. (2012) evaluated an effective removal of iopromide from municipal wastewater using electron beam irradiation technology.

In this study, a hybrid treatment was investigated, which combined the chemical (i.e., photochemical) and conventional biological treatment processes. With this approach, chemical oxidation was used in order to disrupt the original structure of the ICM pollutant in order to make its biodegradation more feasible (Miksch et al. 2015). The aim of our study was therefore to examine the efficiency of ICM removal from synthetic hospital wastewater by UV and UV/TiO_2_ processes and in combination with biological treatment in membrane bioreactors (MBRs). The work was divided into four tasks: (i) estimation of the susceptibility of selected ICM to decay by UV radiation in different aquatic matrices, (ii) determination of optimum hospital wastewater retention time in the UV reactor, (iii) determination of optimal TiO_2_ concentration for the improvement of the effectiveness of UV treatment, and (iv) comparison of the UV and UV/TiO_2_ process effectiveness in the hybrid installation. Three representatives of ICM were selected for the experiments: diatrizoate, iohexol, and iodipamide.

## Materials and Methods

### Chemical Standards

Analytical standards of iohexol, iodipamide, and diatrizoate were purchased from Sigma-Aldrich. The characteristic feature of ICM structure is at least one benzene ring substituted with iodine atoms. Additionally, the aromatic rings are substituted by alkyl side chains coupled to the aromatic ring through amide linkages. The presence of hydroxyl groups improves water solubility of the compounds. In regard to structure, the selected ICM represent to groups: iodipamide and diatrizoate are considered as ionic contrast media, while iohexol is considered as a nonionic contrast medium (see Table 1).

**Table 1:**
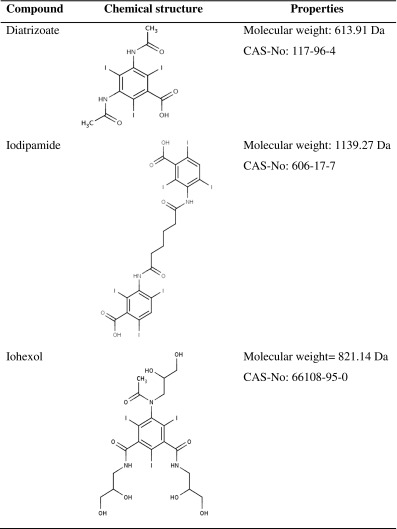
Basic data concerning ICMs used in the studies

Tetrabutylammonium bromide (TBAB) was purchased from Fluka. Titanium oxide (CAS number 1317-70-0) was purchased from Sigma-Aldrich. During the investigation, a powdered anatase form of TiO_2_ (metals basis ≈ 99.8 %) was used.

### Detection of ICM by HPLC

The ICM concentrations were determined by high-performance liquid chromatography (HPLC UltiMate3000, Dionex). Analysis of iodipamide concentration was performed on Hypersil™ GOLD column (Thermo Scientific). Flow rate was set at 0.3 mL min^−1^. The mobile phase was composed of acetate buffer, acetonitryle, and Milli-Q Water in volumetric ratio of 3:42:55. The detection of iodipamide was performed at the wavelength of 238 nm. Analysis of diatrizoate and iohexol concentrations was performed on C8 column (Merck). Flow rate was set at 1.1 mL min^−1^. The mobile phase was composed of phosphate buffer and methanol containing TBAB in volumetric ratio of 85:15. The diatrizoate detection was at the wavelength of 238 nm and the iohexol at the wavelength of 265 nm. The limit of quantification (LOQ), in the case of all the investigated compounds, was equal to 0.2 mg L^−1^. It was established as the second lowest calibration point of their calibration curves (linear regression, *R* ^2^ > 0.98), and the calculated “signal to noise” ratio (S/N) of the compounds was greater than 10. The limits of detection (LODs) of the investigated ICMs were defined when S/N was at the level of 3. LODs in all cases were equal to 0.05 mg L^−1^. The accuracies of the methods were calculated according to ISO 17025, as a recovery. The calculated values of the recoveries were equal to 116.5, 104.4, and 92.4 % for iohexol, diatrizoate, and iodipamide, respectively.

### UV and UV/TiO_2_ Setup

**Fig. 1:**
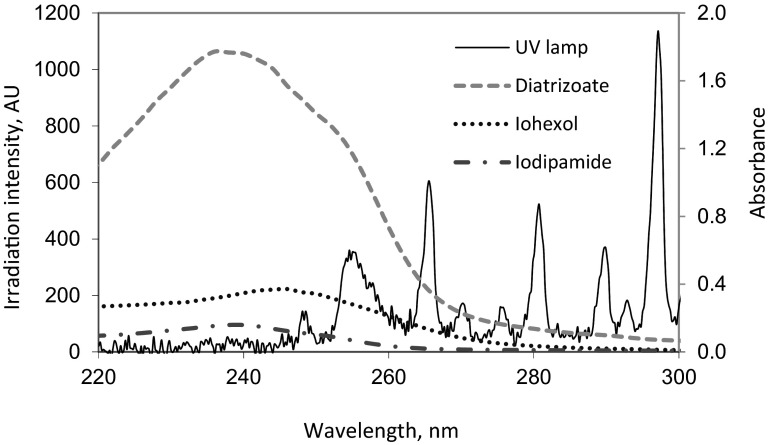
Medium-pressure Hg lamp emission spectrum with reference to absorbance of investigated ICM (water, pH = 7.0)

The UV and UV/TiO_2_ processes were performed in the UV glass reactor of 350-mL volume, equipped with a polychromatic medium-pressure mercury lamp, with emission spectra from 255 to 579 nm (UVI LabP400, Vita Tech, Germany). The lamp emission spectrum was measured by means of Ocean Optic radiophotometer (Fig. 1). The average path length of light through the solution was equal to 10 mm. The light source was characterized with uranyl oxalate as an actinometer according to Miller and Olejnik (2001). The nominal power of UV lamp was equal to 200 W. In the photochemical process experiments, the UV reactor was operated either in a recirculation loop (experiments in Sects. 3.1, 3.2, and 3.3) or as a plug-flow reactor (experiments in Sect. 3.4). The detailed description and the schematic diagram of the UV reactor operated in various flow modes is described in our previous publication (Felis et al. 2009).

### Deionized and Tap Water Experiments

The water composition can affect the photochemical reaction rate, especially the presence of inorganic ions (e.g., bicarbonate and carbonate ions). In order to determine the matrix effect on the photochemical decay of selected ICM, the investigations were conducted in both deionized (Milli-Q Water, Millipore) and tap water (all results in Sect. 3.1). The experiments were performed in water spiked with the analytical standards of iohexol (10 mg L^−1^), diatrizoate (5 mg L^−1^), and iodipamide (10 mg L^−1^). The basic information on composition of tap water used in the experiments is shown in Table 2.

**Table 2:** Basic parameters of the tap water used in photolytic experiments

Parameters	Unit	Value
pH	–	7.4
Thermal conductivity (25 °C)	μS cm^−1^	1118
NH_4_	mg L^−1^	<0.06
NO_2_	mg L^−1^	4.2
NO_3_	mg L^−1^	<0.03
Fe	mg L^−1^	0.0254
Mn	mg L^−1^	0.0159
Total hardness	mg CaCO_3_ L^−1^	458
	mmol L^−1^	4.58
	°dH	25.7

### Synthetic Hospital Wastewater Experiments

The experiments presented in Sects. 3.2–3.4 were performed in synthetic hospital wastewater (average COD of 1.3 g L^−1^), composed of NH_4_Cl (0.250 g L^−1^), KH_2_PO_4_ (0.044 g L^−1^), and CH_3_COONa (1.670 g L^−1^). This hospital wastewater was enriched by iohexol (1.0 mg L^−1^), iodipamide (1.5 mg L^−1^), and diatrizoate (1.0 mg L^−1^) standards.

### Membrane Bioreactors Setup

In this part of the study, two MBRs—control MBR and hybrid MBR—were used. The MBRs were equipped with A4 Size Mat Sheet Membrane, Kubota System (0.4-μm pore size). The working volume of both MBRs was equal to 30 L, and the average concentration of activated sludge in the MBRs was at the level of 4.5 g L^−1^. The sludge retention time was equal to 24 days, with hydraulic retention time maintained at 84 h in both bioreactors. The MBRs were fed with synthetic hospital wastewater, with composition described in Sect. 2.3. The control membrane bioreactor was fed with a synthetic wastewater without photochemical pretreatment. Synthetic hospital wastewater after respectively UV and UV/TiO_2_ treatment (irradiation time = 4 min) was introduced as a feeding medium into the hybrid membrane reactor. The experiments were performed after the sludge adaptation period (72 days), when the sludge retention time has been stabilized.

### Quantum Yields

Kinetic equations were used to quantitatively characterize the UV oxidation. Quantum yields are useful in the design of full-scale installations due to more general nature of the equations. The reaction rates of the ICM photochemical decay is described by Eq. (1), which is a combination of Stark-Einstein and Lambert-Beer law:

1 $$ {r}_{\mathrm{UV}}=-\frac{dC}{dt}=\phi \times {E}_0\times \left(1-{10}^{-\varepsilon \times b\times C}\right) $$

where *r*_UV_ is the initial reaction rate, *C* is the ICM initial (molar) concentration, *φ* is the quantum yield, *E*_0_ is the lamp irradiance, *b* is the average light path into the solution, and *ε* is the weighted average molar extinction coefficient.

After a mathematical transformation of Eq. (1), the values of quantum yields of photochemical decay can be calculated according to Eq. (2):

2 $$ \phi =\frac{r_{\mathrm{UV}}}{E_0\times \left(1-{10}^{-\varepsilon \times b\times C}\right)} $$

The calculations were performed as in the previous study (Felis et al. 2011)—the initial reaction rates (*r*_UV_) were calculated by differentiating exponential curve that fitted experimental points (*C*, *t*) at the correlation factor higher than 0.95. The radiation from the region of 254 to 313 nm was used in all performed investigations, which corresponds to a region where the UV lamp emission (254 to 579 nm) and the absorption spectrum of ICM (200 to 313 nm) overlapped. The acinometric investigations concluded that the lamp irradiance (*E*_0_) was equal to 6.2 × 10^−6^ E L^−1^ s^−1^ (2.46 W L^−1^). The introduction in kinetic considerations of a *weighted average molar absorption coefficient* (*ε*) allowed to determine the actual participation of each wavelength in the absorption spectra of the studied ICM. The *ε* parameter for each ICM was calculated as a weighted average of single molar extinction coefficients determined at selected wavelengths (*λ* = every 2 nm, in the range of active spectrum from 254 to 313 nm).

## Results and Discussion

### Photolysis of Selected ICM in Aquatic Solution with Quantum Yield Determination

This part of the study determined the susceptibility of selected ICM (iohexol, iodipamide, and diatrizoate) to degradation by the polychromatic UV radiation, emitted by the medium-pressure Hg lamp. The absorbance of selected ICM dissolved in water (pH = 7, *t* ≈ 20 °C) with reference to an emission spectra of medium-pressure Hg lamp is presented in Fig. 1. The experiments were performed in deionized (no ions interference with the UV light) and in tap water.

### Photolysis in Deionized Water

**Fig. 2:**
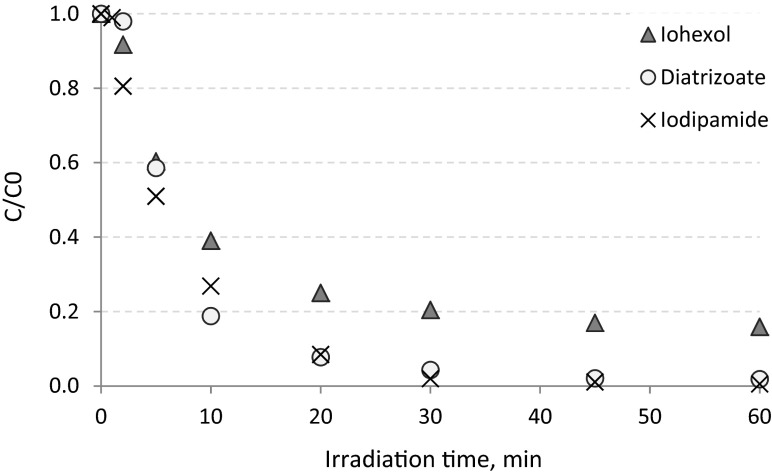
Photolysis of selected ICM in deionized water (pH = 7.0)

The average initial concentrations of ICM during the experiment performed in deionized water were as follows: 11.1, 5.0, and 10.1 mg L^−1^ for iohexol, diatrizoate, and iodipamide, respectively. Among the studied ICM, iohexol was the least efficiently removed in deionized water in the UV process. After 60 min of irradiation, its concentration in reaction solution was equal to 1.8 mg L^−1^, which corresponds to removal of 84 %. Both diatrizoate and iodipamide were more susceptible to the decay by UV radiation (than iohexol), and after 30 min, their concentrations in the solution were at the limit of quantification (LOQ = 0.2 mg L^−1^). This corresponded to removal degrees of 96 and 98 % for diatrizoate and iodipamide, respectively. The removal of selected ICM during direct photolysis performed in deionized water is presented in Fig. 2.

### Quantum Yield Determination

The data used to calculate the quantum yields were taken from the deionized water experiment and are summarized in Table 3. The quantum yields of selected ICM decay in the aqueous solution (pH = 7.0) were established as 0.006, 0.004, and 0.029 for iohexol, diatrizoate, and iodipamide, respectively. This means that iodipamide was the most susceptible to degradation by direct photolysis (as a consequence of light absorption), followed by diatrizoate and by iohexol. The obtained results strongly indicate which of the chemical compounds are most susceptible to this type of degradation; however, the presence of ions in aquatic matrix can affect these yields. Hence, further photodegradation studies were performed in tap water (Sect. 3.4), and ultimately in synthetic hospital wastewater.

**Table 3:** Parameters to calculate the quantum yields of selected ICM decay

Compounds	*C*_0_ (mol L^−1^)	*b* (cm)	*ε* (L mol^−1^ cm^−1^)	*r*_uv_ (mol L^−1^ s^−1^)
Iohexol	1.35 × 10^-5^	1.0	1.71 × 10^4^	1.52 × 10^−8^
Diatrizoate	8.11 × 10^-6^	1.0	1.04 × 10^5^	1.98 × 10^−8^
Iodipamide	8.90 × 10^-6^	1.0	5.54 × 10^3^	1.92 × 10^−8^

### Photolysis in Tap Water

**Fig. 3:**
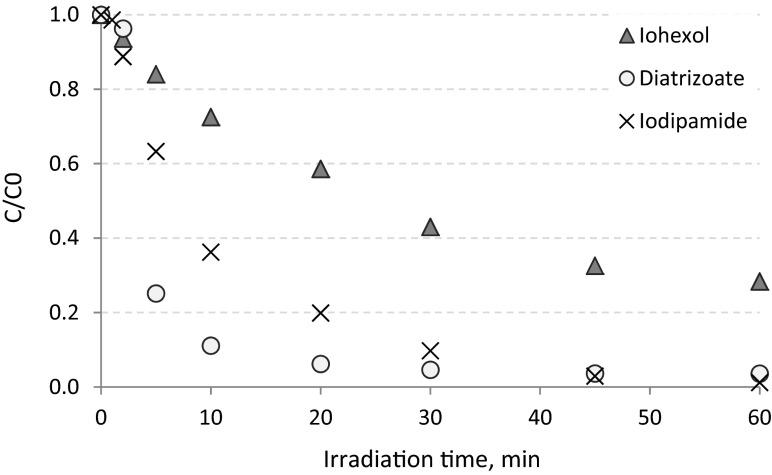
Photolysis of selected ICM in tap water (pH = 7.4)

The removal of selected ICM during direct photolysis performed in tap water is presented in Fig. 3. The solution matrix can influence the photochemical reaction, especially inorganic ions present in the matrix. Inhibitory effects may for instance be caused by carbonate (CO_3_^2−^) and bicarbonate (HCO_3_^−^) anions, which, in combination with the calcium cation, are responsible for the water hardness. The CO_3_^2-^ and HCO_3_^-^ anions can act as radical · OH scavengers and absorb radiation from UV spectrum (especially from UV-A region) which is required for excitation of the target substance (Arslan et al. 2000). The initial concentrations of ICM spiked into tap water were at the same level as in the case of the deionized water experiment, it means 9.6, 5.1, and 10.3 mg L^−1^ for iohexol, diatrizoate, and iodipamide, respectively. Among the investigated substances, the efficiency of iohexol removal was (from investigated compounds) the most sensitive to water matrix composition. After 60 min of direct photolysis performed in tap water, the removal of iohexol was at the level of 72 %. This means that decomposition in tap water was reduced by 12 % compared to the efficiency obtained in deionized water. For diatrizoate and iodipamide a significant influence of the matrix on the photolysis efficiency was not observed. After 30 min of the process, the concentrations of diatrizoate and iodipamide were below LOQ, which means that both substances were removed efficiently.

### Determination of Optimum Retention (Irradiation) Time in UV Reactor

In this study, the optimal retention time of the synthetic hospital wastewater (containing the studied ICM) in the UV reactor was determined. The UV reactor was later used as the pretreatment step before the biological treatment in removal of ICM from the hospital wastewater (hybrid system; Sect. 3.4).

**Fig. 4:**
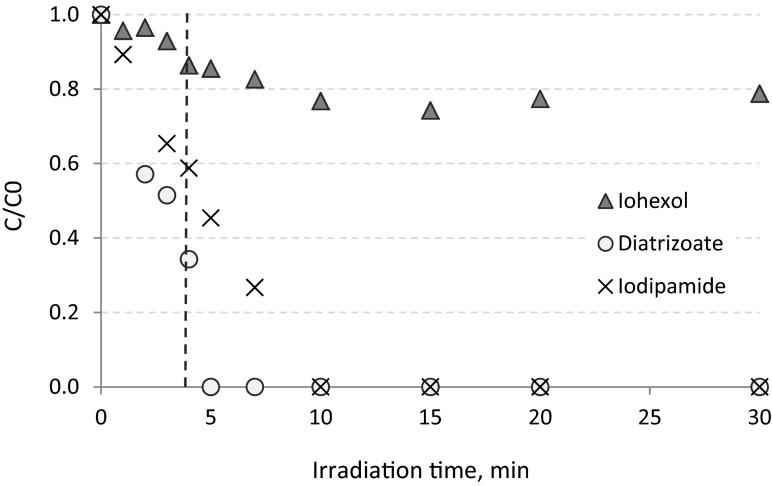
Determination of retention (irradiation) time of hospital wastewater in UV reactor (pH ≈ 7.4)

The solution of synthetic hospital wastewater spiked with ICM was irradiated for 30 min (Fig. 4). After 2 min of UV irradiation, the removal efficiency of iodipamide and iohexol was 7 and 3.5 %, respectively, whereas the diatrizoate was already removed in 43 % (Fig. 4). The complete elimination of diatrizoate occurred after 5 min of the process, whereas the complete elimination of iodipamide was obtained after 10 min. Thirty minutes of the experiment resulted in only 20 % of iohexol decay. For further studies, the optimal media retention time in UV reactor was established as 4 min. Such of value of this parameter caused significant removal of all the investigated compounds, i.e., diatrizoate was removed in 66 %, iodipamide in 41 %, and iohexol in 13 %. Additionally, during such irradiation time no mineralization of organic pollutants was observed—average COD value measured in wastewater before and after UV process remained at the same level (about 1300 mg O_2_ L^−1^). The investigations conducted in synthetic wastewater confirmed that iohexol was the least susceptible to UV radiation and that the wastewater matrix composition (in which the process took place) had a more pronounced adverse effect than in tap water (after 30 min of irradiation 58 % iohexol removal in tap water, compared to only 20 % removal in the synthetic wastewater, Figs. 2 and 3).

### Determination of Optimum TiO_2_ Concentration for UV/TiO_2_ Process

Photocatalytic processes with TiO_2_ addition have been described as an effective method for micropollutant degradation (Doll and Frimmel 2003, 2004; Ternes et al. 2003; Pastrana-Martinez et al. 2012). In order to introduce TiO_2_ as a pretreatment step before the membrane bioreactors, its optimal dose was determined. Three doses of TiO_2_ were selected for the investigation: 100, 300, and 500 mg L^−1^. The first step estimated the ability of ICM adsorption onto the TiO_2_ surface. The ICM solutions (Sect. 2.3) were mixed with suspensions of TiO_2_ (at concentrations of 100, 300, and 500 mg L^−1^). Each experiment lasted 10 min, and the results are summarized in Table 4. Iodipamide was the most efficiently removed from the reaction solution as a result of adsorption onto the surface of TiO_2_. Its removal efficiency was proportional to the applied TiO_2_ dose and contact time. The maximum iodipamide removal as a result of adsorption onto the TiO_2_ surface, which is 17 %, was observed when the applied dose of TiO_2_ was equal to 500 mg L^−1^. In the case of diatrizoate, its removal was insignificant and after 10 min of the experiment with the highest dose of TiO_2_ (500 mg L^−1^) did not exceed the value of 7 %. Iohexol removal due to the adsorption process was at the level of 10 %, regardless of the applied TiO_2_ dose. As reported by Doll and Frimmel (2004), the sorption coefficient may be estimated according to Henry equation, when applied to the low concentration range and in the linear part of the sorption data. This approach was used in our investigation. Thus, the estimated Henry constants, *K*_H_ (pH = 7.4; *T* ≈ 25 °C, TiO_2_ = 0.5 g L^−1^) for the investigated substances were 0.36, 0.29, and 0.68 mg g^−1^ for iohexol, diatrizoate, and iodipamide, respectively. It means that from the investigated compounds, iodipamide had the maximum adsorption capacity onto TiO_2_ surface.

**Table 4:** Sorption of selected ICM onto TiO_2_ surface

TiO_2_ dose (mg L^−1^)	Time (min)	Relative concentration *C*/*C*_0_		
		Iohexol^a^	Diatrizoate^b^	Iodipamide^c^
100	0	1.00	1.00	1.00
	2	0.90	0.98	0.95
	4	0.93	0.99	0.92
	6	0.94	0.99	0.92
	8	0.90	1.00	0.92
	10	0.89	0.97	0.91
300	0	1.00	1.00	1.00
	2	0.91	0.94	0.90
	4	0.00	0.95	0.92
	6	0.91	0.95	0.91
	8	0.93	0.94	0.91
	10	0.89	0.93	0.86
500	0	1.00	1.00	1.00
	2	0.99	0.94	0.91
	4	0.91	0.94	0.90
	6	0.91	0.94	0.90
	8	0.90	0.94	0.88
	10	0.90	0.93	0.83

^a^ *C*_0_ = 1.82 mg L^−1^

^b^ *C*_0_ = 1.06 mg L^−1^

^c^ *C*_0_ = 1.98 mg L^−1^

**Fig. 5:**
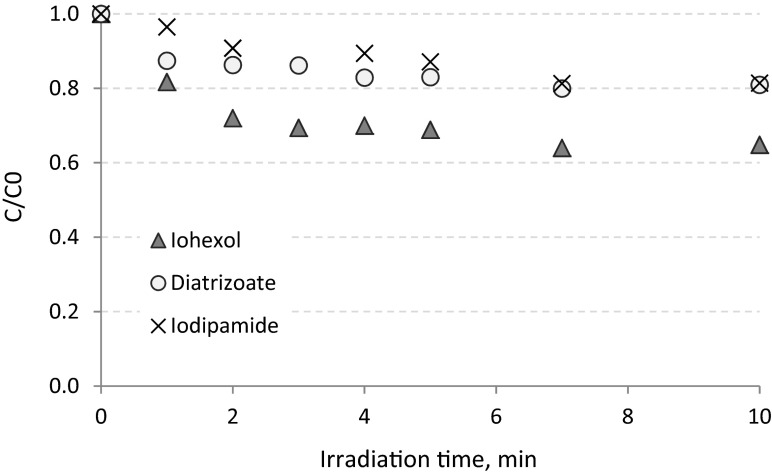
Removal of ICMs from hospital wastewater in UV process enhanced by TiO_2_ in concentration of 100 mg L^−1^

**Fig. 6:**
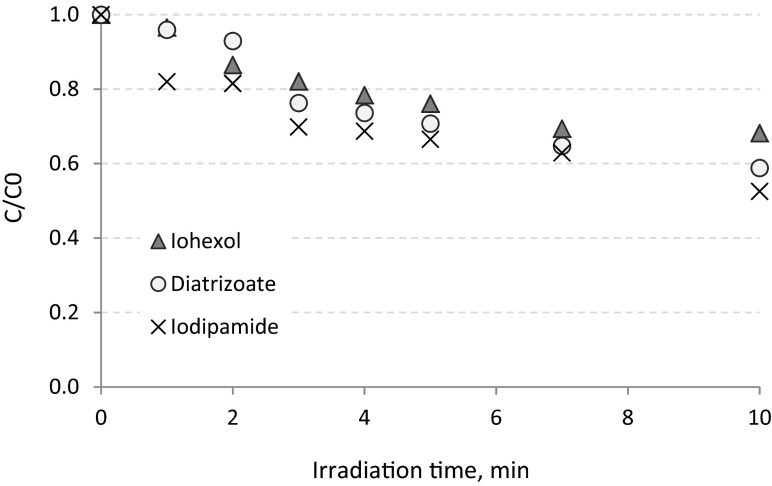
Removal of ICMs from hospital wastewater in UV process enhanced by TiO_2_ in concentration of 300 mg L^−1^

In UV/TiO_2_ processes pollutants may be oxidized by both electron holes generated as a result of TiO_2_ excitation by the UV radiation as well as free radical either at the photocatalyst surface or solution bulk phase (Arslan et al. 2000). During UV/TiO_2_ process with the lowest concentration of TiO_2_ (100 mg L^−1^), iohexol was removed with the highest efficiency, and after 10 min, it was eliminated in 35 %, whereas the diatrizoate and iodipamide decay did not exceed 20 % (Fig. 5). After 4 min of irradiation (Sect. 3.2), iohexol, iodipamide, and diatrizoate were removed from hospital wastewater in 30, 10, and 17 %, respectively. In almost all cases, increasing the concentration of TiO_2_ improved the efficiency of ICM oxidation. After 10 min of irradiation in the presence of 300 mg TiO_2_ L^−1^, diatrizoate was removed twice as effectively as in the presence of 100 mg TiO_2_ L^−1^ (19 and 41 %, respectively) (Fig. 6). Transformation of iodipamide was also more effective—after 10 min of the processes, the decay of almost 50 % was observed. On the other hand, higher concentration of TiO_2_ did not increase the efficiency of the iohexol removal, because after 10 min of the experiment with 300 mg TiO_2_ L^−1^, similar results as in the experiment with 100 mg TiO_2_ L^−1^ was observed.

**Fig. 7:**
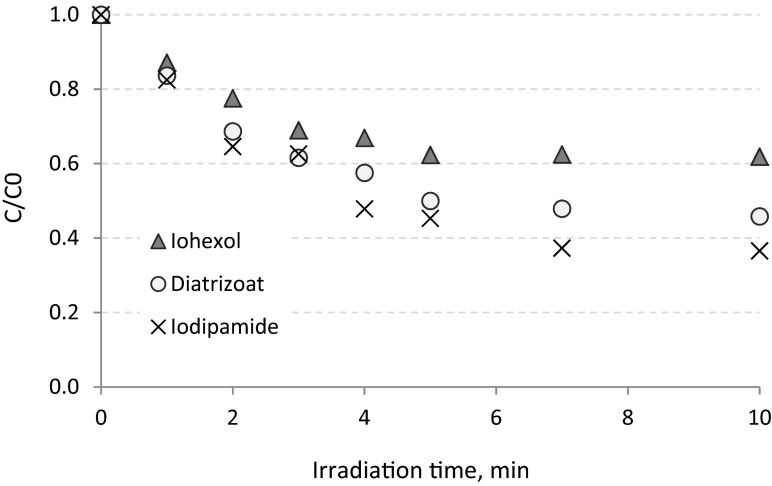
Removal of ICMs from hospital wastewater in UV process enhanced by TiO_2_ in concentration of 500 mg L^−1^

The highest removal efficiency for all the investigated ICM was observed in UV/TiO_2_ process enhanced by dose of 500 mg TiO_2_ L^−1^ (Fig. 7). Significant ICM decay was observed after 4 min of the process—for diatrizoate, it was calculated as 43 %, for iodipamide 52 %, and for iohexol 33 %. After 10 min of the experiment, more than 50 % of the removal was observed for diatrizoate and iodipamide (54 and 63 %, respectively). Iohexol removal (38 %) was at the same level as in the experiments with lower concentration of TiO_2_. According to the obtained data, iodipamide was the most susceptible to degradation in the UV/TiO_2_ process, which was possibly related to its adsorption onto the surface of semiconductor (Doll and Frimmel 2004). Based on the results, it can be also concluded that determining the optimal dose of TiO_2_ may significantly improve the efficiency of the UV/TiO_2_ process.

### ICM Removal from Synthetic Hospital Wastewater in the UV, UV/TiO_2_, and the Hybrid Process

Results below compare the effectiveness of UV and UV/TiO_2_ processes with the hybrid installation, at 4-min retention time in the UV reactor and 500 mg L^−1^ of TiO_2_ in the UV/TiO_2_ process. The hybrid installation combined both photochemical (UV or UV/TiO_2_) and biological processes (MBR), with parameters described in Sects. 3.1–3.3 used in the study. In this part of the study, the UV reactor was operated as plug-flow reactor, contrary to the previous investigations (Sects. 3.2 and 3.3), where it was operated in a recirculation loop. The results on the ICM removal obtained in the preliminary tests (with UV and UV/TiO_2_ only) differed from those obtained in the lab-scale hybrid installation, which means that the different modes of UV reactor operation could influence the removal efficiency of the target substances.

**Fig. 8:**
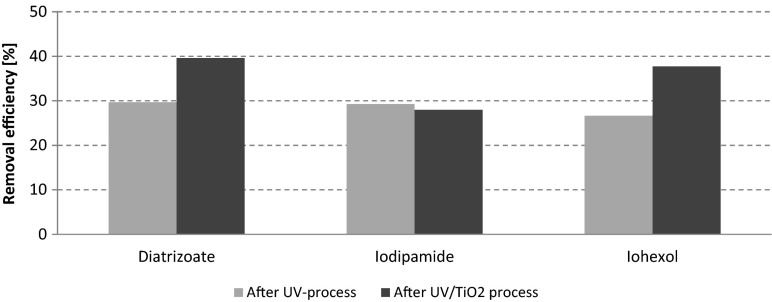
Comparison of effectiveness of UV and UV/TiO_2_ processes in ICM removal from hospital wastewater (plug-flow UV reactor, irradiation time = 4 min)

During this experiment, the removal efficiency of diatrizoate was higher in the UV/TiO_2_ experiments than in the UV experiments and equaled to 40 and 30 %, respectively. In the case of iohexol, 27 % of this compound was removed in the UV process. Addition of 500 mg TiO_2_ L^−1^ in the UV/TiO_2_ process improved the iohexol decay to 38 %. No significant differences in removal efficiency of iodipamide during UV and UV//TiO_2_ processes were observed—the removal efficiency was equal to 29 and 28 %, respectively, in UV and UV//TiO_2_ processes (Fig. 8). The obtained results showed that not in every case the addition of TiO_2_ to the UV process significantly improved the efficiency of ICM decomposition.

**Fig. 9:**
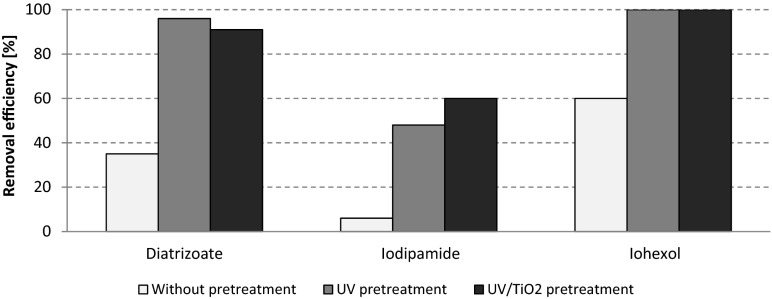
Comparison of effectiveness of ICM removal from hospital wastewater by means of MBR combined with various types of photochemical pretreatment

The results from combined photochemical and biological treatment (Fig. 9) show that the use of photochemical pretreatment (UV or UV/TiO_2_) significantly improved the ICM elimination in the biological treatment step. The removal efficiencies in the biological step without pretreatment equaled to 38 % for diatrizoate, 6 % for iodipamide, and 60 % for iohexol. With pretreatment (regardless whether only UV or UV/ TiO_2_ were used), more than 90 % elimination of diatrizoate was obtained, while iohexol was removed completely (100 %). For iodipamide, which was practically previously nonbiodegradable, application of the UV radiation as pretreatment before MBR caused its 50 % degradation, while application of the UV/TiO_2_ process increased its elimination efficiency to 60 %. However, no significant differences in removal efficiency was observed between the UV treatment alone or in combination with the TiO_2_ process (Fig. 9), indicating that the wastewater matrix played a role in diminishing the TiO_2_ particles efficiency. In summary, inserting the photochemical processes as a pretreatment step before the biological treatment significantly improved the removal of ICM, compared to the photochemical or to the biological processes alone.

## Conclusions

The experiments showed that elimination of selected ICM from water can be successfully conducted with the photochemical UV and UV/TiO_2_ processes. During the experiments, the quantum yields of ICM decay in ideal aqueous solution (deionized water, pH = 7.0) were established as 0.006, 0.004, and 0.029 for iohexol, diatrizoate, and iodipamide, respectively. This means that iodipamide was the most susceptible to degradation by direct photolysis (UV) induced by the polychromatic irradiation. However, iohexol was the most efficiently degraded in the photochemical UV/TiO_2_ process. More importantly, UV and UV/TiO_2_ processes were successfully applied as a pretreatment step before a biological wastewater treatment stage (hybrid methods). The photochemical processes disrupted the chemical structure of the studied ICM and this enables them to the further transformation in the biological stage of treatment. Such combination of the photochemical and biological processes significantly enhanced the removal efficiency of the studied ICM from the synthetic hospital wastewater in comparison with the removal efficiency obtained in the biological processes without photochemical pretreatment. It means that the aforementioned processes can be a promising tool for the removal of the iodinated contrast media from the aquatic environment.

## Abbreviations

CAS: Chemical abstracts service
COD: Chemical oxygen demand
ICM: Iodinated contrast media
HPLC: High-pressure liquid chromatography
LOQ: Limit of quantification
MBR: Membrane bioreactor
SBR: Sequencing batch reactor
UV: Ultraviolet
WWTP: Wastewater treatment plant
